# Supplementary material for: Industrial agglomeration and air pollution: A new perspective from enterprises in Atmospheric Pollution Transmission Channel Cities (APTCC) of Beijing-Tianjin-Hebei and its surrounding areas, China
Source: PLoS One. 2021 Jul 23;16(7):e0255036. doi: 10.1371/journal.pone.0255036 (PMC8302251; doi:10.1371/journal.pone.0255036)
Supplement: Attachment — Submitted filename: Response to the Editors and reviewers20210601.docx [file pone.0255036.s003.docx]

## **Response to the Editors and reviewers**

**1. Responses to the Editors:**

**Comment (1):**

*If applicable, we recommend that you deposit your laboratory protocols in protocols.io to enhance the reproducibility of your results. Protocols.io assigns your protocol its own identifier (DOI) so that it can be cited independently in the future. For instructions see: http://journals.plos.org/plosone/s/submission-guidelines#loc-laboratory-protocols.*

**Response:** Thank you for your kind advice. We will deposit this protocol in future. We have also modified and improved the manuscript according to the reviewers’ comments, with a particular focus on improving language.

**Comment (2):**

*Please review your reference list to ensure that it is complete and correct. If you have cited papers that have been retracted, please include the rationale for doing so in the manuscript text, or remove these references and replace them with relevant current references. Any changes to the reference list should be mentioned in the rebuttal letter that accompanies your revised manuscript. If you need to cite a retracted article, indicate the article’s retracted status in the References list and also include a citation and full reference for the retraction notice.*

**Response:**

Thank you for your suggestion. We have reviewed the reference list and ensured that we have not cited papers that have been retracted.

**Comment (3):**

*Is the manuscript technically sound, and do the data support the conclusions? The manuscript must describe a technically sound piece of scientific research with data that* *supports the conclusions. Experiments must have been conducted rigorously, with appropriate controls, replication, and sample sizes.* *The conclusions must be drawn appropriately based on the data presented.*

*Reviewer #1: Yes*

*Reviewer #2: Partly*

**Response to Reviewer #2:**

We are very much appreciative of the time you have taken to review our revised manuscript and provide valuable comments. The relationship among enterprises’ agglomeration, pollution emissions, and air quality is complex; as such, there may be factors that cannot be reflected in the data. There are various methods to measure agglomeration; we believe that EGI and HHI are appropriate and have provided rationales to this end. The data were examined and the data quality was guaranteed, and the sample size was sufficient. From our limited understanding, we believe the manuscript is technically sound, and the data can support the conclusions. The manuscript also provides some novel examples and perspectives that we would really like to share with other researchers.

**Comment (4):**

*Is the manuscript presented in an intelligible fashion and written in standard English?*

*PLOS ONE does not copyedit accepted manuscripts, so the language in submitted articles must be clear, correct, and unambiguous. Any typographical or grammatical errors should be corrected at revision, so please note any specific errors here.*

*Reviewer #1: Yes*

*Reviewer #2: No*

**Response to *Reviewer #2*:**

Thank you for your kind advice and comments. We appreciate the time you have put into reviewing the manuscript. The manuscript has been edited by a Language Editing Service to ensure that the submitted article is clear, correct, and unambiguous to readers. We have uploaded the language editing certificate to the attachments.

**Comment (5):**

*The modified version is clearer than the original, but proofread might be helpful to improve scientific writing. Please check all the article again. For example,*

*Line1-13, Font inconsistency with other parts of article.*

*Line 42, Line 88, Word choice of "externalities", "impact" would be better.*

*Line 45, generates more "pollutants", "pollution" would be better.*

*Line 47, "production concentration", The usage seems weird, and would be better to change it.*

*Line 48, 545, word choice of "spillover effect", Would it be better to replace a word or not used so many times.*

*Line 51-54, 399, “Beijing-Tianjin- Hebei (BTH) Collaborative Development Plan", “Opinions on Strengthening the Construction of the Key Platform for the Transfer of the Beijing-Tianjin-Hebei (BTH) Industry”, "Blue-Sky Protection Campaign" would be better written in italics.*

*Line 63, emission intensity per unit of land area, "per square kilometers" would be better.*

*Line 82-83, word choice of "agglomeration", "pollution aggregation" would be better.*

*Line 103, word choice of "pollutant emission", "pollution" would be better.*

*Line 114, Some Literature, "L" should not use capitalized.*

*Line 119, Some literature shows, It should be plural and no "s".*

*Line 239, Spatial correlation analysis versus Line 438, Spatial autocorrelation test, Uniform usage is better.*

*Line 349-350, word choice of "high concentration of enterprises", "agglomeration" would be better.*

*Line 389, word choice of "heaviest", "highest" would be better.*

*Line 398-399, word choice of "ultra-low transformation", "lowest" would be better.*

*Line 498/ 521/ 533, what are the meaning of 10%, 5%, and 1%? How about the p-value?*

*Line 537, word choice of "metropolis", "city" would be better.*

**Response:**

Thank you for reviewing the language issues in this manuscript. We have checked the manuscript again, and have revised much of the article, whereby details of this article have been improved. The revised manuscript has been edited by a Language Editing Service to ensure that the submitted article is clear, correct, and unambiguous to readers. We have uploaded the language editing certificate in the attachments. Thank you again for your careful proofreading and comments.

**Comment (6):**

*it would be better to combine 1. Introduction and 2. Literature review and makes the article look concise.*

**Response:**

Thank you for your kind advice and comments. We have revised the literature review and Introduction sections and combined them to provide a more concise introduction.
